# The Fate of Linear DNA in *Saccharomyces cerevisiae* and *Candida glabrata*: The Role of Homologous and Non-Homologous End Joining

**DOI:** 10.1371/journal.pone.0069628

**Published:** 2013-07-24

**Authors:** Mary W. Corrigan, Christine L. Kerwin-Iosue, Alexander S. Kuczmarski, Kunj B. Amin, Dennis D. Wykoff

**Affiliations:** Department of Biology, Villanova University, Villanova, Pennsylvania, United States; Institut de Genetique et Microbiologie, France

## Abstract

*In vivo* assembly of plasmids has become an increasingly used process, as high throughput studies in molecular biology seek to examine gene function. In this study, we investigated the plasmid construction technique called gap repair cloning (GRC) in two closely related species of yeast – *Saccharomyces cerevisiae* and *Candida glabrata*. GRC utilizes homologous recombination (HR) activity to join a linear vector and a linear piece of DNA that contains base pair homology. We demonstrate that a minimum of 20 bp of homology on each side of the linear DNA is required for GRC to occur with at least 10% efficiency. Between the two species, we determine that *S. cerevisiae* is slightly more efficient at performing GRC. GRC is less efficient in *rad52* deletion mutants, which are defective in HR in both species. In *dnl4* deletion mutants, which perform less non-homologous end joining (NHEJ), the frequency of GRC increases in *C. glabrata*, whereas GRC frequency only minimally increases in *S. cerevisiae*, suggesting that NHEJ is more prevalent in *C. glabrata*. Our studies allow for a model of the fate of linear DNA when transformed into yeast cells. This model is not the same for both species. Most significantly, during GRC, *C. glabrata* performs NHEJ activity at a detectable rate (>5%), while *S. cerevisiae* does not. Our model suggests that *S. cerevisiae* is more efficient at HR because NHEJ is less prevalent than in *C. glabrata*. This work demonstrates the determinants for GRC and that while *C. glabrata* has a lower efficiency of GRC, this species still provides a viable option for GRC.

## Introduction

**Figure 1:**
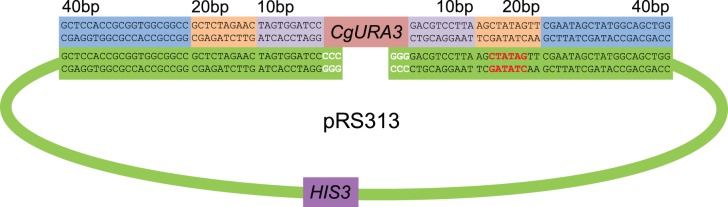
Schematic of GRC including selected base pair homologies. GRC takes advantage of base pair homology between the linearized vector pRS313 and *CgURA3* PCR product. pRS313 was cut with the restriction enzymes *Sma*I (indicated by white text) and *Eco*RV (indicated by red text). Based on the primer sets used to amplify *CgURA3*, the *CgURA3* PCR product to be inserted had either 40 bp, 20 bp, 10 bp, or a combination of homologies.

Plasmid construction is an essential technique in molecular biology. Assembly of plasmids, containing specific DNA fragments, can be carried out either *in vitro* or *in vivo*. *In vitro* cloning, which requires restriction enzymes and DNA ligase, can be costly and inefficient for high throughput methods. *In vivo* cloning, such as gap repair cloning (GRC), utilizes homologous recombination (HR) activity and can be cheaper and more efficient [1], [2]. GRC uses available homologous ends of a linearized vector and a DNA fragment (usually generated by PCR) to fuse the two, creating a circular plasmid (Figure 1). Yeast species are appealing for GRC as many species appear to perform HR efficiently [3], [4], [5], [6], [7]. By understanding the minimal requirements for GRC, costs can be minimized.

Several studies have examined GRC in the budding yeast, *Saccharomyces cerevisiae*, and demonstrated this species is capable of efficient GRC [1], [8], [9], [10]. These analyses specify that 30 base pairs of homology is sufficient for DNA integration into a linearized vector, but it may not be the minimum requirement [9], [11]. Recently, a similar study indicated that GRC is a viable cloning technique in *Schizosaccharomyces pombe* [4]. Additionally, our laboratory routinely uses GRC in *C. glabrata*, but the specific requirements for efficient GRC are unknown.

Our studies with *C. glabrata* suggest that HR appears to be less efficient than in *S. cerevisiae*, as deletion of genes using HR is less efficient in *C. glabrata* [5](data not shown). We expected that a detailed analysis of GRC in the two species would elucidate the role of HR, the mechanism by which this technique takes place, and non-homologous end joining (NHEJ). Additionally, *C. glabrata* is closely related to *S. cerevisiae* and pathogenic to mammals, allowing for comparisons over evolutionary time. We examined the role of two genes involved in HR and NHEJ – *RAD52* and *DNL4*. *RAD52* has previously been identified as a gene involved in DNA double-strand break repair and it facilitates HR in *S. cerevisiae* and *S. pombe* [12]. When *RAD52* is deleted, HR should be decreased and we would expect that GRC will either not take place or will be dramatically reduced. *DNL4* is required for NHEJ, which is the repairing of double stranded DNA breaks without homologous ends via its ligase activity [13], [14]. Mutations in *dnl4^+^* in *S. pombe* lead to increased frequency of HR, and we predicted that loss of *DNL4* should lead to higher frequency of GRC in *S. cerevisiae* and *C. glabrata*, as NHEJ would be reduced.

The goal of this study was to define the determinants of GRC in both *S. cerevisiae* and *C. glabrata*, with regard to the amount of homology and ratios of DNA concentrations. Additionally, we aimed to examine the role of HR in GRC and how NHEJ influences the frequency of GRC. Finally, we incorporated our data into a model of the fate of linear DNA when transformed into the two species.

## Materials and Methods

### Yeast Strains and Growth Conditions

Wild-type *S. cerevisiae* and wild-type *C. glabrata* were used as the host strains for GRC transformations as both are *ura3* ^−^ *his3* ^−^ (See Table 1 for strains). Deletions of *RAD52* and *DNL4* in both *S. cerevisiae* and *C. glabrata* were generated using antibiotic resistance genes *KANMX6* and *NATMX6* (conferring resistance to G-418 and nourseothricin, respectively) and homologous recombination to delete the ORFs [5], [15], [16], which was confirmed by PCR. For transformations, yeast strains were grown in YEPD medium at 30°C until logarithmic growth phase (OD_600_ 0.2–0.5). To select for plasmids, strains were grown in synthetic dextrose (SD) medium with CSM lacking the appropriate amino acids (either histidine or uracil) (Sunrise Science, San Diego, CA, USA). Transformations were performed using a standard lithium acetate protocol [17], [18], [19].

**Table 1:** Strains used in this study.

Strain	Genotype	Reference
***S. cerevisiae***		
EY57 (DC3)	K699 with *MAT* **a**	Wykoff and O'Shea (2001)
DC153	*rad52*Δ *NATMX6* in DC3	This study
DC152	*dnl4*Δ *NATMX6* in DC3	This study
***C. glabrata***		
BG99 (DG5)	*his3*Δ (1+631)	Cormack and Falkow (1999)
DG74	*ura3*Δ *NATMX6* in BG99	Kerwin and Wykoff (2012)
DG173	*rad52*Δ *NATMX6* in DG74	This study
DG172	*dnl4*Δ *NATMX6* in DG74	This study

### DNA for Transformations

*CgURA3* was amplified from *C. glabrata* wild-type DNA with varying amounts of homology on each side using a mixture of *Taq* and *Pfu* polymerase (Figure 1). To amplify *CgURA3* with 40 bp of homology, primers with the sequences: 5′-GCTCCACCGCGGTGGCGGCCGCTCTAGAACTAGTGGATCCtgacttttacactaatgagg and 3′-GGTCGACGGTATCGATAAGCTTGATATCGAATTCCTGCAGctagatattacatgcataac were used, where the lowercase letters correspond to the *CgURA* gene. For 20 bp and 10 bp of homology, truncated versions of these primers were used. pRS313 (*HIS3^+^*) [20] was linearized with *Sma*I or with *Eco*RV and *Sma*I. PCR products and linear vectors were subjected to gel electrophoresis, purified using a Geneclean II Kit (M.P. Biomedicals, CA, USA), and quantified with a NanoDrop 2000. The *CgURA3* PCR products and linearized vectors were co-transformed at different molar ratios (0, 0.02, 0.2, and 1.0) into *S. cerevisiae* and *C. glabrata* strains. Based on the size of fragments, we estimated that equal amounts of DNA in nanograms were a molar ratio of 0.2, as the plasmid was ∼5× the size of the insert. For all transformations, we used 50 ng of linearized vector. All cells from *S. cerevisiae* transformations and 20% of cells from *C. glabrata* were plated onto SD medium lacking histidine, so that individual colonies per plate were <500 cfu. After 3 days of growth, these *HIS3^+^* colonies were then replica plated to SD medium lacking uracil.

### Sequencing

To sequence plasmids created by GRC, cells were grown overnight in selective medium and yeast plasmids were purified utilizing an ammonium acetate procedure [19]. Yeast plasmid preparations were transformed into chemi-competent XL1-Blue *Escherichia coli* cells and plasmids were isolated by an alkaline lysis protocol. We were successful in isolating plasmid DNA from yeast >90% of the time. Plasmids were sequenced (GENEWIZ, NJ, USA) with T7 and T3 primers to determine the sequence on each side of the insertion.

## Results

### Investigation of GRC Efficiency in *S. cerevisiae* and *C. glabrata*

**Figure 2:**
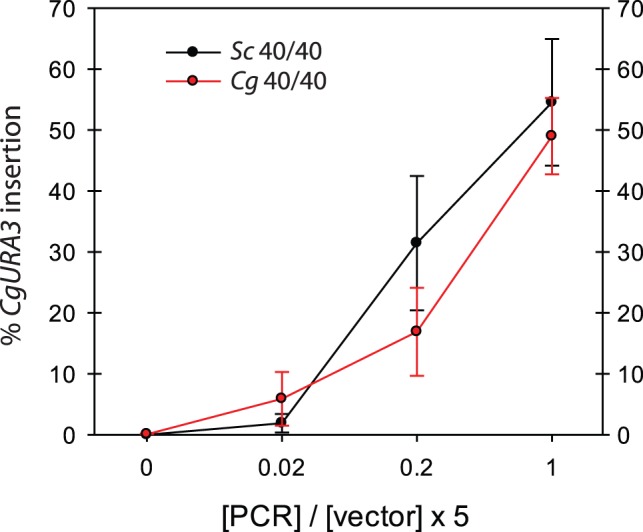
Average insertion percentage for *CgURA3* with 40 bp homology in *S. cerevisiae* and *C. glabrata*. Cells were co-transformed with *CgURA3* PCR product that had 40 bp of homology at an increasing molar ratio of PCR to vector. Colonies from GRC experiments in both wild-type yeast species were plated on medium lacking histidine and subsequently replica plated to medium lacking uracil. Percentages of *CgURA3* insertion into linearized pRS313 (*HIS3* ^+^) were calculated and averaged for at least 3 independent experiments (n = 3) and the error bars are s.e.

To compare the ability of *S. cerevisiae* and *C. glabrata* to carry out GRC, we performed transformation reactions using the same preparations of linearized vector, pRS313 (*HIS3^+^*), and the same *C. glabrata* gene, *CgURA3* with promoter, in both species. pRS313 contains an autonomously replicating sequence (ARS) that is functional in both species and *CEN6* from *S. cerevisiae* [19], [20]. It is unclear whether *CEN6* is functional in *C. glabrata*, but it is unlikely as there are very specific sequence requirements for centromeric sequences in *C. glabrata* [21], [22] and we have observed relatively quick plasmid loss under non-selective conditions in *C. glabrata* (data not shown). Each *CgURA3* product was amplified to include 40 bp of vector homology on each side, which is sufficient homology for GRC. Using different molar ratios of *CgURA3* to digested pRS313 (0, 0.02, 0.2, and 1.0), we titrated the concentration of DNA required for effective GRC. We measured GRC efficiency as the percentage of *HIS3^+^*colonies (from linearized pRS313) that became *URA3^+^* (Figure 2). Not surprisingly, the higher the molar ratio of insert DNA (*CgURA3* with homology) to digested vector, the better the GRC efficiency. We determined that an insert to vector molar ratio of 1.0 produced an average 55% DNA insertions in *S. cerevisiae* and an average 49% DNA insertion in *C. glabrata*, and adding 5-fold molar excess of insert DNA allowed GRC to approach 90% in both species (data not shown). We concluded that *S. cerevisiae* and *C. glabrata* both efficiently engage in GRC, and that GRC can occur even when there is a relatively low amount of insert. It is not surprising that equal molar amounts are not required for efficient GRC, as it is likely that multiple DNA fragments enter the cell during transformation, and all that is required for our assessment of GRC is for one linear DNA vector molecule to be repaired by one insert with homologous ends.

It is worth noting that an alternative reason for observing *URA3^+^ HIS3^+^* colonies could be integration of either piece of DNA into the genome or *URA3^+^* integration coupled with re-circularization of the pRS313. To confirm that we were observing GRC plasmids, we grew 92 Ura^+^His^+^ colonies from *S. cerevisiae* and 115 colonies from *C. glabrata* on non-selective (YEPD) medium overnight to allow for plasmid loss and then replica-plated to medium containing 5-FOA, which selects against cells that are expressing the *URA3^+^* gene (Figure S1 and Figure S2). In *C. glabrata* 113/115 colonies resulted in FOA^R^ colonies, and in *S. cerevisiae* 91/92 colonies, suggesting almost all Ura^+^ colonies were a consequence of *URA3^+^* incorporation into a plasmid as opposed to stable integration into the genome. Importantly, all of the FOA^R^ colonies except for one *C. glabrata* colony resulted in a His^−^ phenotype, indicating that the vast majority of *URA3* and *HIS3* genotypes are coupled on a plasmid and not a consequence of chromosomal integration events.

### Determination of Minimal Base Pair Homology for Detectable GRC Results

**Figure 3:**
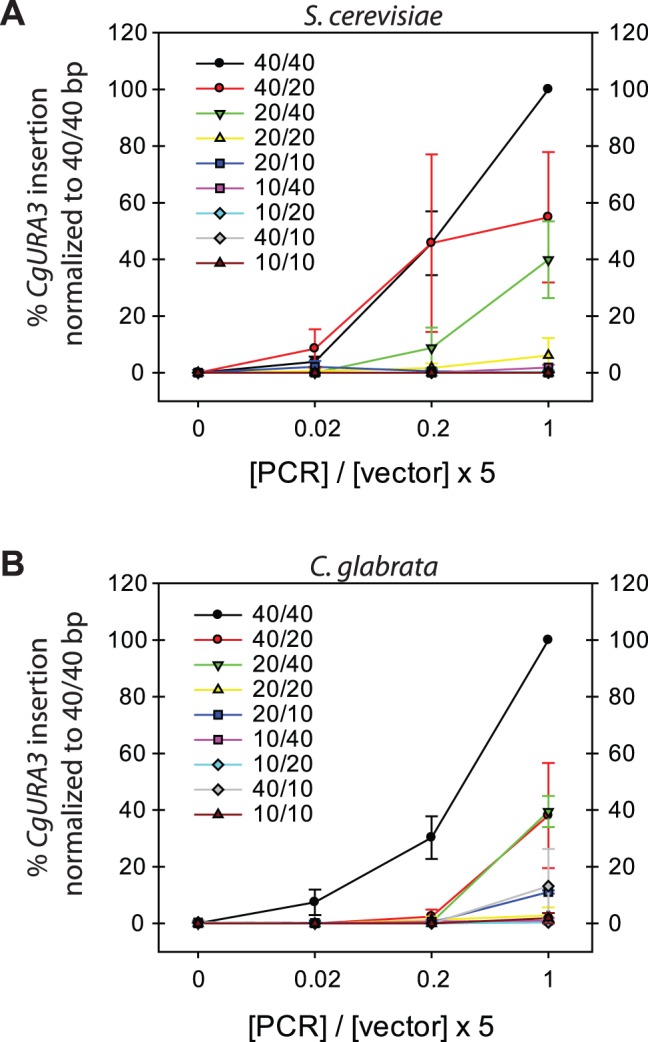
Average insertion percentages of *CgURA3* with selected bp homologies in *S. cerevisiae* (A) and *C. glabrata* (B). Results from GRC experiments using wild-type strains and varying amounts of base pair homologies. Transformants were plated on medium lacking histidine and subsequently replica plated to medium lacking uracil. Percentages of *CgURA3* insertion into linearized pRS313 (*HIS3* ^+^) were calculated from three independent experiments where 40/40 bp of homology was set at 100% in each experiment and errors are s.e.

To investigate the minimal amount of homology required for GRC, we performed transformation reactions in both *S. cerevisiae* and *C. glabrata* with varying amounts of homology on each side of the *CgURA3* PCR product: 40 bp, 20 bp, and 10 bp (Figure 3A and 3B). We determined that decreasing the homology on both sides from 40 bp to 20 bp dramatically decreased the GRC efficiency. In both *S. cerevisiae* and *C. glabrata*, decreasing the homology from 40 base pairs to 20 base pairs on a single side reduces the efficiency by 50%–60%. Further decreasing the homology from 40 bp to 10 bp on both sides allowed for only extremely rare GRC activity. From this we conclude that decreasing the homology on a single side of the DNA insert significantly affects the efficiency of GRC. Furthermore, the data are not consistent with the hypothesis that one side of high homology (40 bp) can compensate for low homology (10 bp) on the other side – i.e. the 40/10 PCR products perform poorly relative to the 20/20, even though this is more total homology with the 40/10 PCR products, suggesting that there is not a simple counting mechanism for homologous recombination. Our data suggest that the minimum requirement for homology is 20 bp on each side and that each side of homology has an individual role in GRC. These requirements are similar in both *S. cerevisiae* and *C. glabrata*.

**Figure 4:**
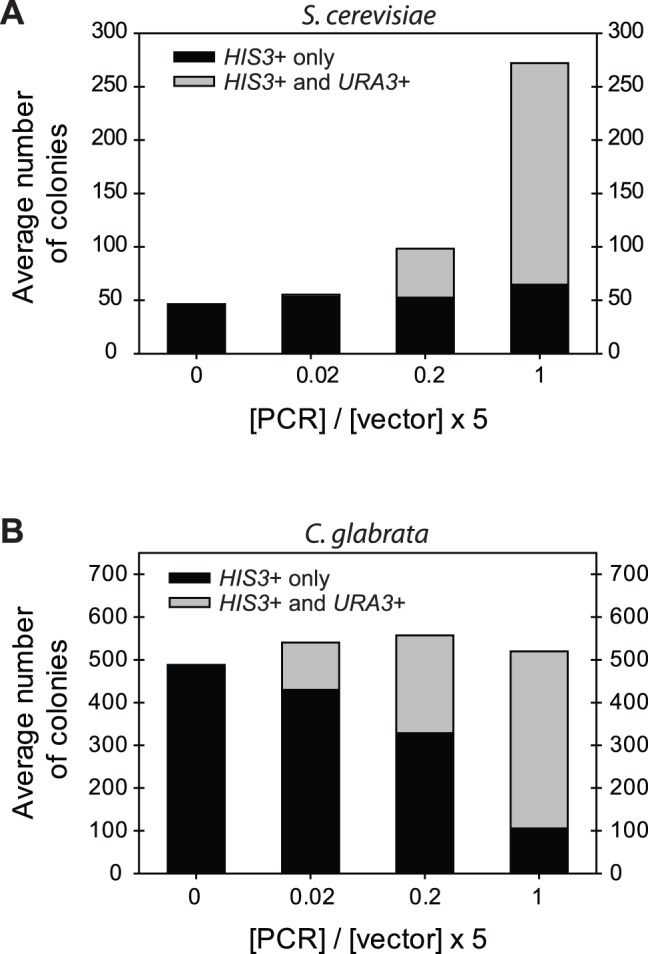
Average number of colonies with *CgURA3* inserted into pRS313 (*HIS3* ^+^) in *S. cerevisiae* (A) and *C. glabrata* (B). Average number of wild-type strain colonies from GRC experiments using *CgURA3* PCR product that has 40 bp of homology on each side. Averages were calculated for each of the different molar ratios of PCR to vector. Average number of colonies containing pRS313 (*HIS3* ^+^) only and those containing the vector (*HIS3* ^+^) with inserted *CgURA3* are shown. These averages are from 3 separate experiments.

While performing these experiments, we noticed a different trend between the two species. When transforming 40 bp of homology on each side, the number of colonies produced by *S. cerevisiae* increased consistently as the insert to vector ratio increased from 0.0 to 1.0 (Figure 4A); however, when transforming *C. glabrata* with 40 bp of homology on each side, the number of colonies produced remained relatively constant, despite the increasing insert to vector ratio (Figure 4B). These data are consistent with a difference in HR activity vs. NHEJ activity in the two species. If HR is more active in *S. cerevisiae* relative to *C. glabrata*, then we would expect only GRC clones to be observed when insert is added. If NHEJ is more active, as in *C. glabrata*, then there should be approximately the same amount of colonies observed regardless of insert to vector ratio. Our data suggest that NHEJ activity, the mechanism by which a vector re-circularizes, with or without insert, is more prevalent in *C. glabrata* than it is in *S. cerevisiae*.

### Effects of Inactivating Homologous Recombination or Non-homologous Recombination Pathways on the Frequency of GRC

To examine the role of HR and NHEJ on the rate of GRC, we deleted *RAD52* and *DNL4* in both *S. cerevisiae* and *C. glabrata* and repeated the transformation experiments with 40/40 bp and 40/20 bp of homology. *RAD52* is a key gene in genetic recombination and DNA repair, and is required for most forms of HR, but deleting *RAD52* does not completely eliminate HR [7], [12], [23], [24]. *DNL4* also mediates DNA repair, but through non-homologous repair mechanisms [14], [25].

**Figure 5:**
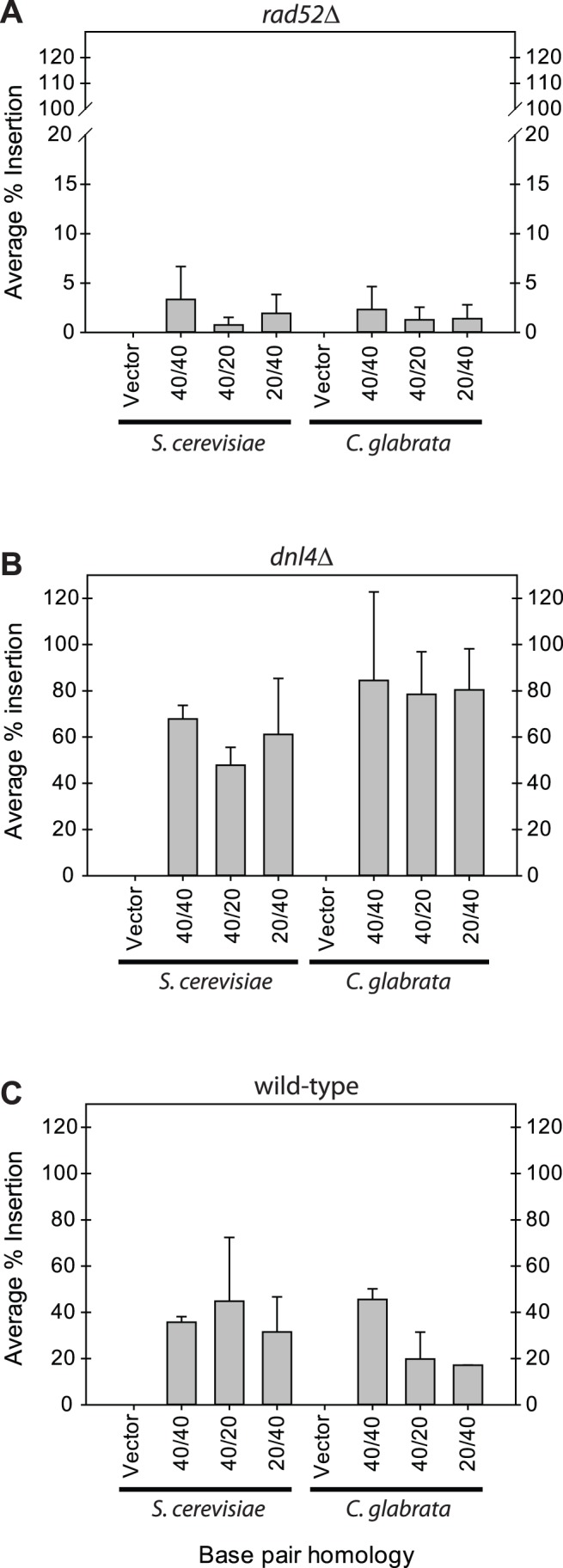
Average insertion percentage of *CgURA3* into pRS313 (*HIS3* ^+^) in *rad52*Δ (A), *dnl4*Δ (B), and wild-type (C) strains in *S. cerevisiae* and *C. glabrata*. Average insertion percentage from GRC experiments using *CgURA3* PCR products that have either 40 bp of homology on each side or a combination of 40 and 20 bp of homology. PCR to vector molar ratio was constant at 1. Average insertion percentage was calculated as *URA3* ^+^/*HIS3* ^+^ and vector had no added PCR product. Note difference in scale for A relative to B and C.

Decreasing HR activity through deletion of *RAD52* considerably reduces the frequency of GRC in both species compared with wild-type strains (Figure 5A and 5C – note difference in scale). In *S. cerevisiae*, the data support that deleting *RAD52* does decrease the frequency of GRC, but does not completely eliminate it. Likewise, in the *Cgrad52*Δ strain, the efficiency of GRC is dramatically decreased but not completely eliminated.

Inactivating the pathway responsible for NHEJ activity has different effects on *S. cerevisiae* and *C. glabrata* (Figure 5B and 5C). Notably, in *Scdnl4*Δ cells, the percent of DNA insertion into the plasmid is relatively unaffected compared with transformations in the wild-type - i.e. loss of NHEJ does not dramatically increase GRC efficiency. However, in *C. glabrata*, GRC increases in a *Cgdnl4*Δ strain, suggesting that NHEJ is more active, and loss of NHEJ drives the equilibrium of re-circularization towards HR.

### Determination of Proportion of Resealed Vectors through HR or NHEJ in *S. cerevisiae*

**Figure 6:**
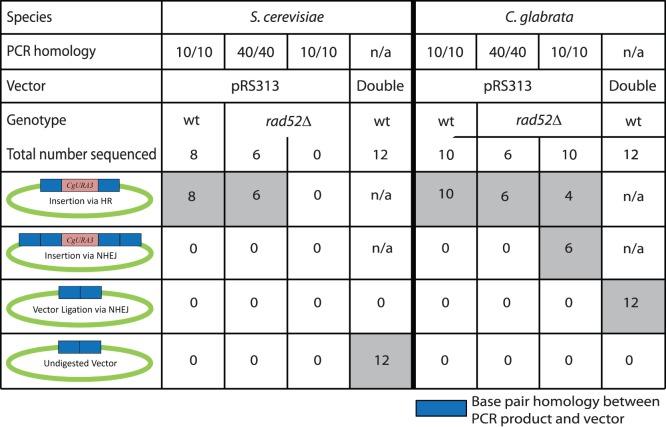
Outcomes of sequencing plasmids from GRC experiments. Sequences from rescued plasmids showed one of four different results: insertion via HR, insertion via NHEJ, vector ligation via NHEJ, or undigested vector. Each column represents a different GRC experiment with varying species, PCR homology, vector, and genotype. The two vector types include pRS313 (digested with only *Sma*I) and a doubly digested pRS313 (*Sma*I and *Eco*RV). The plasmids from the doubly digested vector were rescued from colonies that were *HIS3* ^+^ and *URA3* ^−^. The values given represent the number of plasmid sequences displaying each outcome out of the total number of plasmids sequenced. The shaded regions highlight outcomes.

In some transformations, such as those in a *rad52*Δ strain or where PCR product with low bp homology was used, the likelihood of acquiring plasmids with the *URA3* insertion was low. To determine whether these rare *URA3^+^* transformants were a consequence of HR or NHEJ, we rescued the plasmids in *E. coli* and sequenced the plasmids that resulted from the transformations. Not surprisingly, plasmids rescued from a *S. cerevisiae* wild-type transformation with 40 bp of homology on each side exhibited re-circularization via HR (data not shown); however, even with 10 bp of homology on each side, *S. cerevisiae* wild-type appeared to only re-circularize via HR (Figure 6). Likewise, in a *Scrad52*Δ, which has decreased HR (Figure 5A), transformations with 40 bp of homology on each side yielded plasmids that re-circularized via HR, indicating that loss of *RAD52* does not completely eliminate GRC.

We sequenced re-circularized *S.cerevisiae* plasmids that did not contain *URA3* (*HIS3* ^+^ only), and found that all appeared to not have been digested at all with *Sma*I. Knowing that restriction enzyme digestion may not proceed to completion, we hypothesized that these few *HIS3* ^+^ *URA3* ^−^ plasmids could be a consequence of incomplete digestion. To test this hypothesis, we doubly-digested pRS313 with two blunt cutting enzymes, *Eco*RV and *Sma*I, expecting that if re-circularization is caused by NHEJ and not a consequence of partial restriction enzyme digestion, we should observe the loss of 13 bp in the plasmid sequence (Figure 1). We only observed completely undigested plasmid in *S. cerevisiae* plasmids, suggesting either we are unable to observe NHEJ in *S. cerevisiae* in this experiment, or that a singly digested plasmid ligated with no change in sequence. Supporting the lack of detectable NHEJ in this GRC assay, we were unable to recover *URA3^+^ HIS3^+^* plasmids in *S. cerevisiae* strains where *RAD52* was deleted and the homology was decreased to 10 bp on each side.

### Determination of Proportion of Resealed Vectors through HR or NHEJ in *C. glabrata*

In *C. glabrata* we also rescued the plasmids from the rare *URA3^+^ HIS3^+^* transformants using DNA inserts with 10 bp of homology on each side or from *Cgrad52*Δ transformations. The sequencing data from these plasmids indicated that DNA was inserted via HR in *C. glabrata* wild-type (with 10 bp of homology) and in *Cgrad52*Δ (with 40 bp of homology), strengthening the conclusion that HR is the preferred mechanism in *C. glabrata* as well (Figure 6). We then transformed the *Cgrad52*Δ strain using linear vector and *CgURA3* with 10 bp of homology, and noted a difference between *S. cerevisiae* and *C. glabrata*. Whereas we never identified a *URA3^+^ HIS3^+^* colony in *Scrad52*Δ with 10 bp homology, we identified colonies that were both *URA3* ^+^ and *HIS3* ^+^ in *C. glabrata*. We purified these plasmids and confirmed that 60% of the time the linear PCR product was inserted into the plasmid via NHEJ, because there was a 10 bp duplication of sequence flanking both sides of the *URA3* ^+^ gene.

Following this experiment, plasmids were rescued from a transformation using a doubly digested plasmid and no insert. In *S. cerevisiae*, 100% of the sequences from these double-digested plasmids indicated that the vector was likely never fully digested. In *C. glabrata*, 100% of the sequences demonstrated that the rescued vectors were digested and re-circularized, lacking the 13 bp, and thus were a consequence of NHEJ. These data reveal that *C. glabrata* is more capable of performing NHEJ relative to *S. cerevisiae*.

Our results may appear to conflict with other published studies – i.e. our observation of no NHEJ activity in *S. cerevisiae*; however, it is worth pointing out four major differences between this study and the others. First, we used at least 40× less plasmid DNA (50 ng vs. 2 µg) than another study using plasmids making it unlikely that we have saturated the HR machinery in the cell and skewing results towards HR [13]. We chose these low concentrations to see subtle differences between the two species in a titration of different variables, but these conditions minimize NHEJ activity (Figure 2), as previous experiments have demonstrated less than 5 transformants/µg of non-homologous DNA [26]. Second, most NHEJ studies examine events in the chromosome, not in plasmids [5], [26], [27]. Third, many studies indicate high levels of HR in yeast species and so it is not surprising given our conditions that we are not observing relatively rare NHEJ events [10], [13], [24]. Finally, previous studies have indicated that direct ligation may be a mode of repair, but we have also examined the role of partial plasmid digestion (see below). Because we digest with two blunt end restriction enzymes, and observe ligation events in *C. glabrata*, but not in *S. cerevisiae,* we can conclude that this ligation/NHEJ activity is higher in *C. glabrata* relative to *S. cerevisiae*. Therefore, in our conditions we do not observe NHEJ in *S. cerevisiae,* but there are likely rare NHEJ events that we are not observing. In fact, in other studies even with higher concentrations of plasmid in *S. cerevisiae* the vast majority of “re-circularization events” were actually incomplete digestion events [10].

### Assessment of the Impact of Chromosomal Integration on GRC

A possible complication to our work is that *URA3* ^+^ could be integrated into the chromosomal genome via HR or NHEJ at the same time that vector re-circularizes, overestimating the frequency of GRC. To determine whether integration into the genome of the *URA3* ^+^ gene could cause a Ura^+^ phenotype independent of plasmid re-circularization, we performed a reciprocal experiment. We transformed both species as before, but selected for *URA3^+^* first and measured the frequency of these colonies that were *HIS3* ^+^. In *S. cerevisiae*, we did not observe any Ura^+^ colonies in the absence of linearized vector and we only observed Ura^+^ His^+^ colonies during co-transformation with both PCR product and linearized vector, suggesting little/no NHEJ.

In *C. glabrata*, we observed Ura^+^ colonies with PCR product alone; however, the frequency of these colonies was ∼5% of the maximal number of colonies observed when linearized vector was present. Additionally, during co-transformation, we observed ∼50% of colonies that were Ura^+^ but not His^+^. Initially, this might suggest that NHEJ and chromosomal integration is very high; however, the *CgURA3* PCR product is derived from *C. glabrata*, and we hypothesized that HR between the PCR product and the endogenous *Cgura3*::*NATMX6* locus might be occurring in these transformations. Because we deleted *CgURA3* in the genome with the *NATMX6* cassette, we expected loss of the *NATMX6* cassette if *CgURA3* is integrated into the genome through HR. Importantly, all Ura^+^ His^+^ colonies were resistant to nourseothricin and all Ura^+^ His^−^ colonies were sensitive, indicating that HR is far more prevalent than NHEJ in *C. glabrata*.

## Discussion

The goal of this project was to characterize GRC in *S. cerevisiae* and *C. glabrata*. Our results suggest that GRC can be effectively carried out in both yeast species. This has previously been demonstrated in *S. cerevisiae* but not in *C. glabrata*. We were also able to uncover the determinants of GRC for both species. Our data indicate that 20 bp of homology on each side of the PCR product is required for detectable GRC to occur. An insert:vector molar ratio of 1.0 was shown to be sufficient for obtaining insertion of the PCR product at ∼50%, while increasing this ratio to 5.0 drives GRC to almost 100%. We also found that *S. cerevisiae* is slightly more efficient at performing GRC. This result was supported by the GRC data we obtained using the *rad52*Δ mutants. The *S. cerevisiae rad52*Δ mutant was only able to perform GRC at a very low frequency and required 40 bp of homology on each side, indicating that this species is very dependent on HR for re-circularization. The *C. glabrata rad52*Δ mutant produced re-circularized plasmids that contained *URA3* at a low frequency as well. However, the sequencing data suggests that NHEJ, in addition to HR, is an option for inserting *URA3* into the vector (Figure 6). The presence of NHEJ activity in *C. glabrata* may be an effect of slower DNA degradation in this species, although we have not tested that hypothesis in this work. Interestingly, sequencing from a separate experiment (data not shown) indicated that *C. glabrata* displayed a strong preference for performing HR. In this experiment, a sequence contained a fragment of salmon sperm DNA that had been inserted into the vector by GRC, with only 11 bp of homology on one side of the salmon sperm DNA fragment. This fortuitous finding suggests DNA may persist longer in *C. glabrata,* allowing more time for NHEJ or extremely rare HR events to occur, even with unintended targets, such as small fragments of carrier salmon sperm DNA.

The *S. cerevisiae dnl4*Δ mutant shows a similar rate of GRC as the wild-type strain. Here again, our data suggest that *S. cerevisiae* may not be capable of performing NHEJ at a detectable rate in this GRC assay, indicating HR is the only means to re-circularize DNA through insertion. In contrast to *S. cerevisiae*, the *Cgdnl4*Δ strain shows an increased frequency of GRC compared to wild-type, suggesting that NHEJ activity is detectable in this GRC assay and that *C. glabrata* has more NHEJ activity in general.

**Figure 7:**
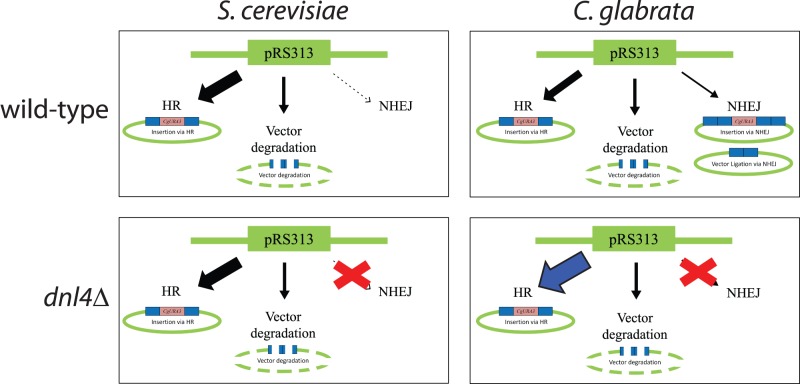
Model of the fate of linear DNA in *S. cerevisiae* and *C. glabrata*. In *S. cerevisiae*, we were unable to observe re-circularization via NHEJ; however, it is likely that there is still a low level of NHEJ activity. Consequently, there is little change in the frequency of HR in both *S. cerevisiae* wild-type and *dnl4*Δ. In contrast, in *C. glabrata*, we observe insertion and vector ligation via NHEJ. Deletion of *DNL4* results in an increase in HR, indicating that NHEJ is more prevalent in *C. glabrata*.

Based on results from all of the experiments that were performed, we generated a model for the fate of linear DNA in *S. cerevisiae* and *C. glabrata* (Figure 7). In *S. cerevisiae*, it appears that the linear DNA (both pRS313 and PCR product) takes two out of three avenues in the majority of instances: HR or vector degradation (the eventual fate of transformed linear DNA in cells if vector is not re-circularized). Removing NHEJ activity in *S. cerevisiae*, through deletion of *DNL4*, has little effect on GRC, because there is little NHEJ to begin with. On the other hand, the fate of linear DNA in *C. glabrata* is influenced by HR, NHEJ, or degradation. When NHEJ activity is deleted in *C. glabrata*, there is an increase in HR. Although both species are capable of performing GRC at a high level, this model suggests that *S. cerevisiae* is the preferred species in which to perform this technique. Our data indicate that NHEJ is more prevalent in *C. glabrata*; however we cannot fully determine whether this is because NHEJ is inherently more active in this species, or whether linear DNA is degraded more slowly in *C. glabrata* and the NHEJ machinery is able to work for a longer period.

## Supporting Information

Figure S1 ***S. cerevisiae*** **Ura^+^His^+^ cells are primarily a consequence of GRC.** Using 40 bp homology on each side, colonies that were Ura^+^His^+^ were picked to a YEPD plate and then replica-plated to 5-FOA plates and colonies were scored for growth. Then, the colonies were replica-plated to medium lacking uracil or histidine to assess whether the two markers were coupled on a plasmid.
(EPS)

Figure S2 ***C. glabrata*** **Ura^+^His^+^ cells are primarily a consequence of GRC.** The same experiment was performed as described in Figure S1. One colony out of the 113 was identified as His^+^ and Ura^−^ and is potentially a consequence of plasmid integration into the genome.
(EPS)
